# Supplementary figures and images for: Corticosterone pattern-dependent glucocorticoid receptor binding and transcriptional regulation within the liver
Source: PLoS Genet. 2021 Aug 10;17(8):e1009737. doi: 10.1371/journal.pgen.1009737 (PMC8378686; doi:10.1371/journal.pgen.1009737)

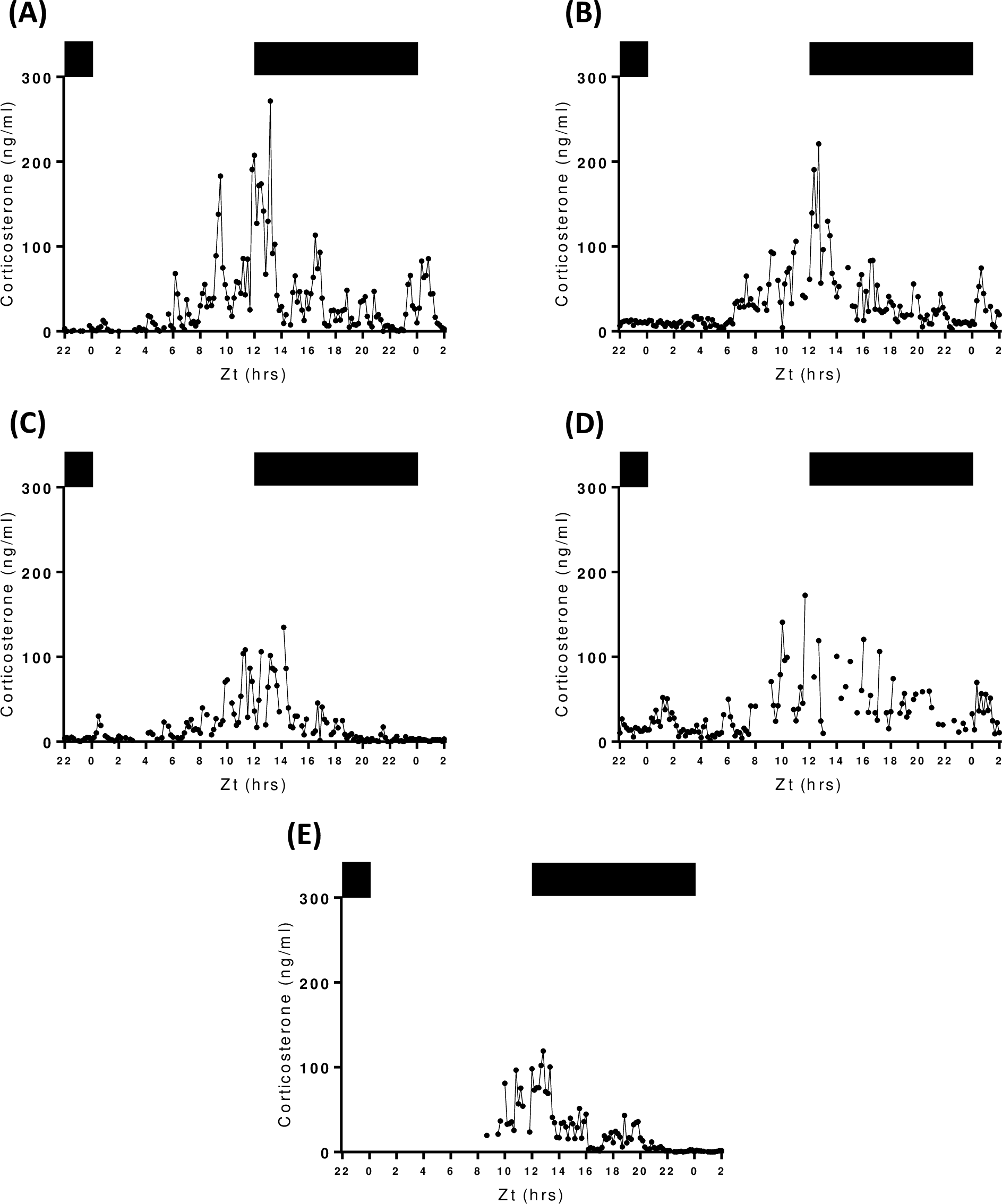

Supplement: S1 Fig — (A-E) Blood samples were collected every 10 min over a 24-hour period from rats and total CORT levels measured in the plasma by radioimmunological assay. Light schedule was 12:12 (on Zt 0 / off Zt 12) with dark phase indicated by shaded bar along the top of the plot. (TIF) [file pgen.1009737.s001.tif]

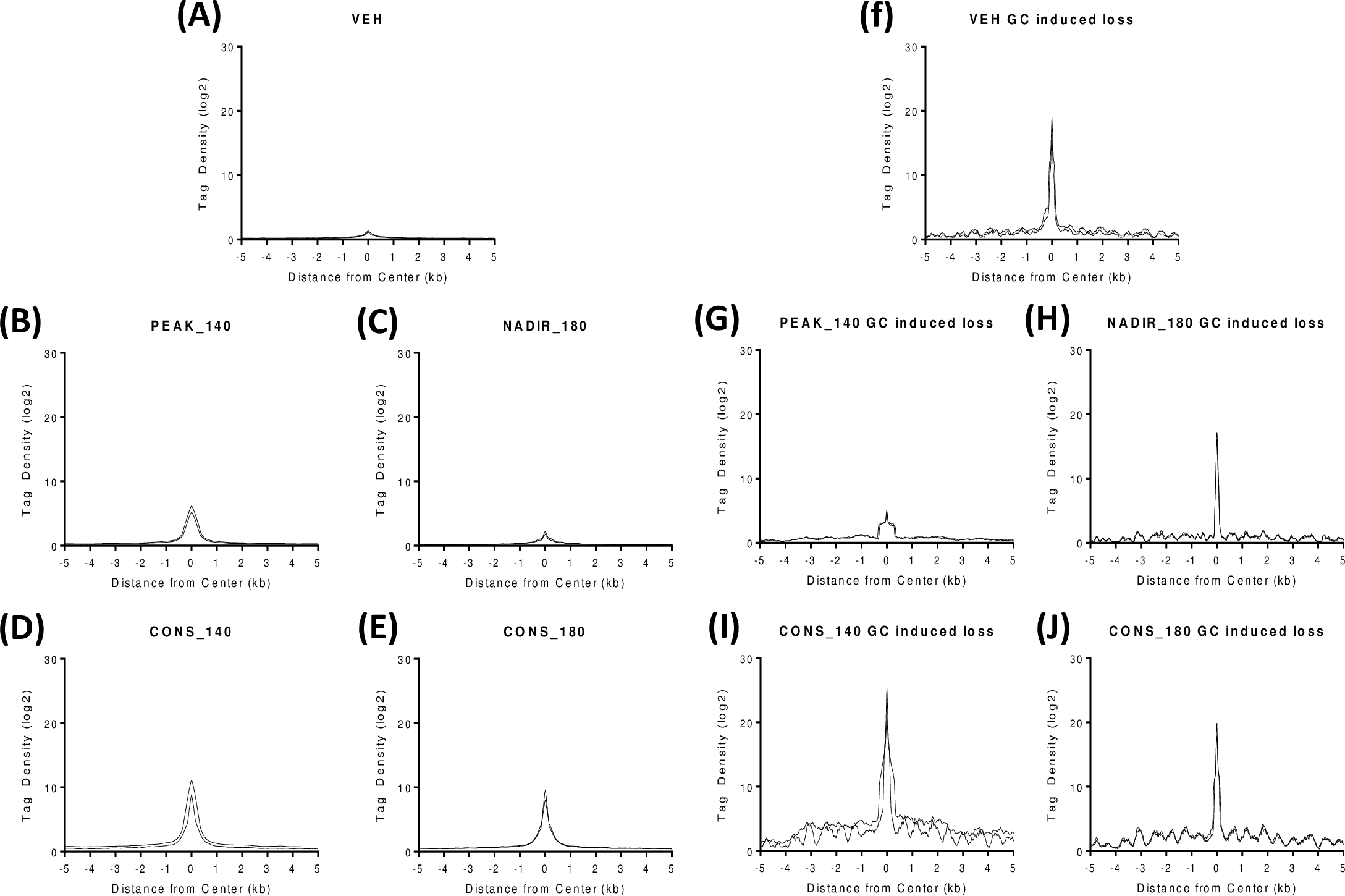

Supplement: S2 Fig — (A-E) Tag density at identified GR enriched regions across conditions was plotted across a series of histograms in response to (A) VEH, (B) PEAK_140, (C) NADIR_180, (D) CONS_140 and (E) CONS_180. (F-J) Histograms of tag density were also plotted at sites with reduced enrichment in response to CORT infusion compared to VEH. In all cases, for (F) VEH, (G) PEAK_140, (H) NADIR_180, (I) CONS_140 and (J) CONS_180, robust peaks in tag density were localised at enrichment centre and reduced to negligible levels within 1 kb in either direction. Raw tags (log2) of each replicate (N = 2) were segmented in 5 b bins, spanning 5 kb in each direction from enrichment centre. (TIF) [file pgen.1009737.s002.tif]

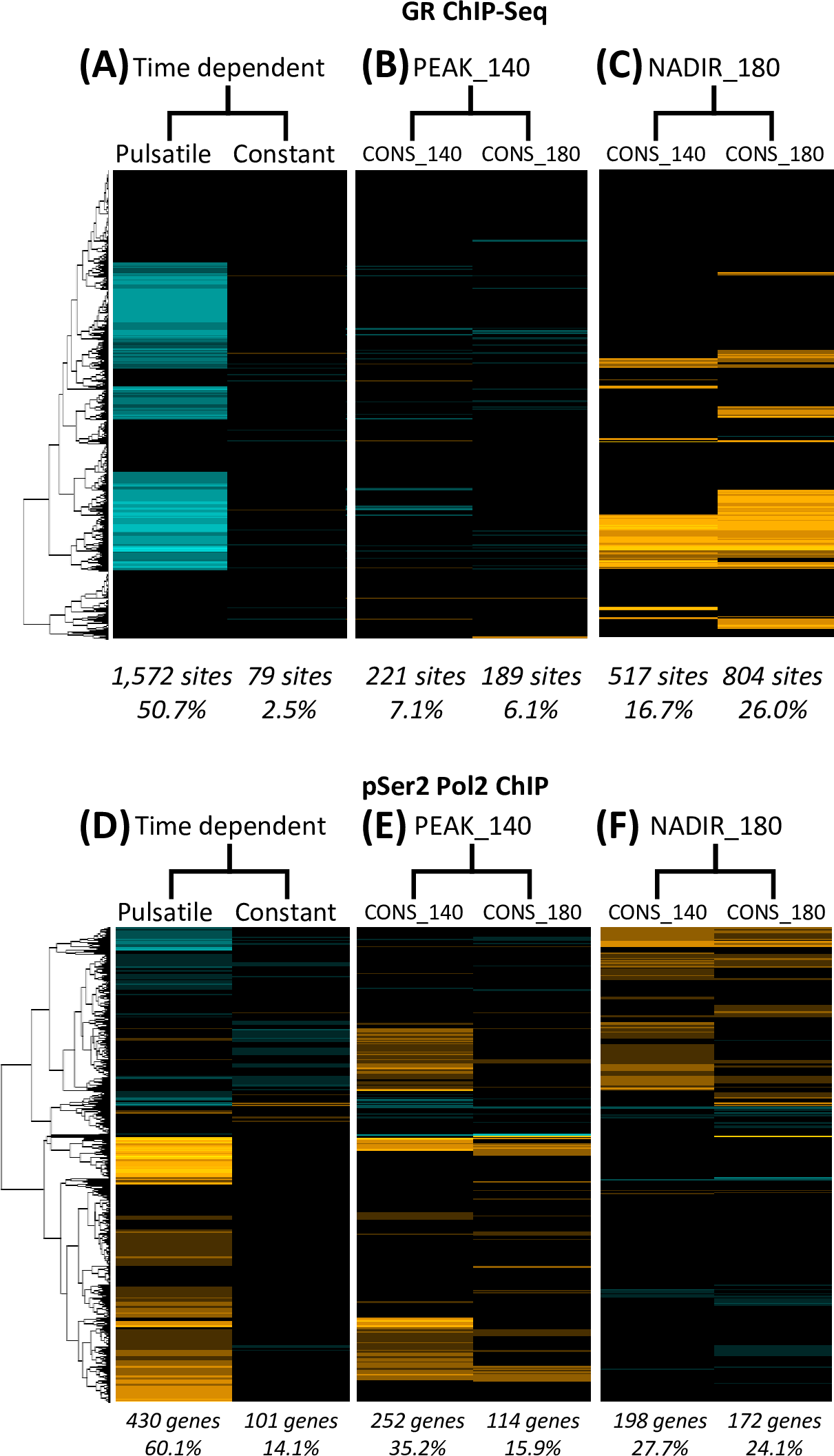

Supplement: S3 Fig — Results visualised within these heatmaps were assessed within differentially enriched regions to VEH in response to the indicated pulsatile or constant CORT infused time point. (A) GR time dependent sites were defined as sites enriched to VEH in response to pulsatile or constant CORT, that were also significantly regulated between 140 min and 180 min CORT infused time points. Of those, fold change is visualised for 140 min relative to 180 min time points for both pulsatile and constant CORT infusion. (B and C) GR sites identified as differentially enriched relative to VEH were assessed in significant fold change comparisons, with (B) PEAK_140 fold changes in enrichment visualised in comparison to CONS_140 and CONS_180 and (C) NADIR_180 fold changes in enrichment visualised in comparison to CONS_140 and CONS_180. (D) pSer2 Pol2 time dependent sites were defined as sites that had been identified as significantly differentially enriched relative to VEH, then found to have a significantly difference between 140 min and 180 min, with fold change in 140 min relative to 180 min visualised here for both pulsatile or constant CORT infusion. (E and F) pSer2 Pol2 sites differentially enriched to VEH by (E) PEAK_140 and (F) NADIR_180. In both cases, fold changes in enrichment are visualised at these sites compared to CONS_140 and CONS_180. Heatmap indicates merged replicate tags (N = 2), colour intensity indicates degree of fold change compared to VEH as shown within the -8 to 8 scale bar and regions of interest clustered by fold changes hierarchically clustered by Euclidean distances. (TIF) [file pgen.1009737.s003.tif]

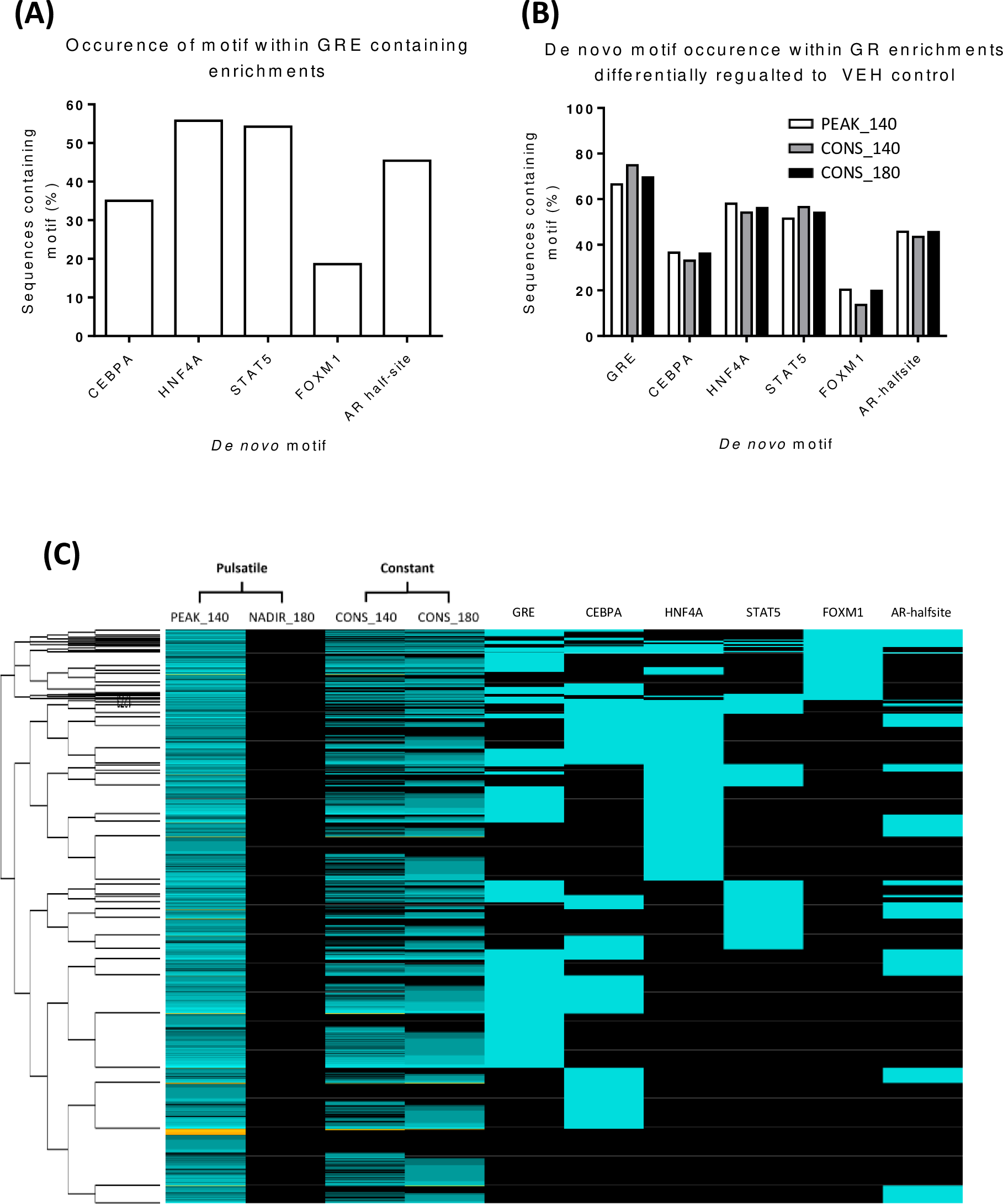

Supplement: S4 Fig — (A) Bar graph displays the top 5 most significant motifs discovered within GR bound, GRE containing regions across all CORT infused time-points. (B) Motifs identified in GR binding regions that were enriched by PEAK_140 (white), CONS_140 (grey) or CONS_180 (black) time points compared to VEH. (C) Lanes 1–4 of heatmap indicates fold change in GR enrichment within identified binding regions in the liver in response to either PLS or CONS at 140 min and 180 min compared to VEH. Data presents merged replicate tags (N = 2) hierarchically clustered according to fold change (log2) and colour intensity indicates degree of fold change as indicated by the scale bar between -8 to 8 (top left). Lanes 5–10 indicate the presence (blue) or not (black) of the motif indicated at the top of the lane within the GR enriched site (horizontal row of heatmap). De novo motifs were identified within 233 b regions in either direction from the centre of identified GR enriched regions. (TIF) [file pgen.1009737.s004.tif]

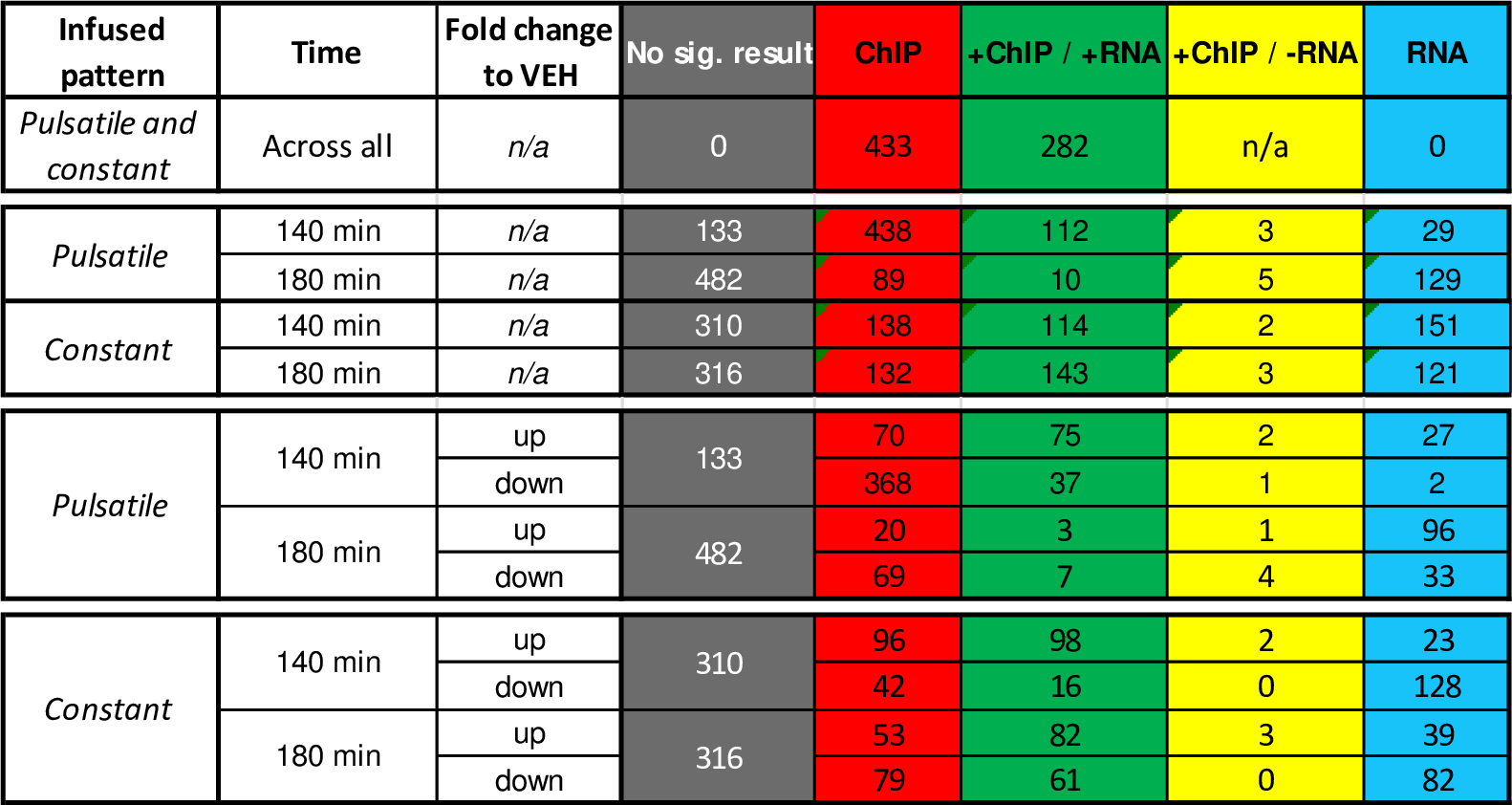

Supplement: S5 Fig — Of the 715 differentially pSer2 Pol2 occupied genes; results in the top row (row 1) are split into differentially occupied pSer2 Pol2 ChIP only (red), as well as genes that were differentially occupied and expressed within the RNA-Seq data set (green) across all CORT infused conditions. Rows 2–5 split data by 140 min and 180 min time points in response to pulsatile or constant CORT infusion and whether the increased or decreased fold change was observed in both data sets (green) or were opposingly increased / decreased (yellow), the number of non-significant ChIP results (black) and genes whose RNA was differentially expressed without an associated significant ChIP result (blue). Rows 6–9 split data by up-regulated and down-regulated pSer2 Pol2 occupation at PEAK_140 and NADIR_140 and RNA expression at 180 min whilst rows 10–13 split data for constant CORT infusion in the same manner. Data represents fold changes > 1.5 and adjusted p-value < 0.05. (TIF) [file pgen.1009737.s005.tif]

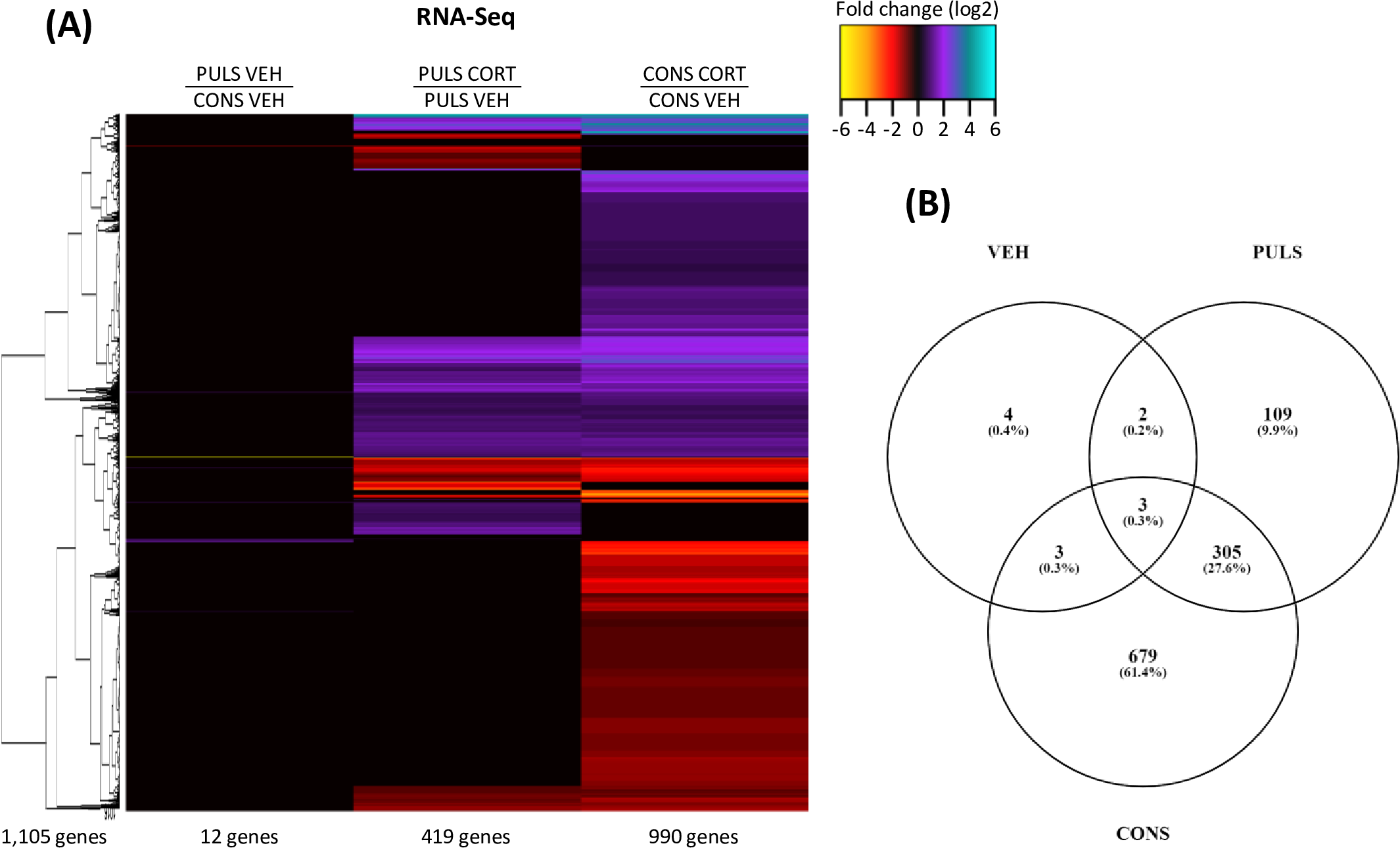

Supplement: S6 Fig — (A) Fold changes of 1,105 differentially expressed exonic RNA transcripts (bottom left) was visualised for pulsatile vehicle compared to constant vehicle (left), pulsatile CORT compared to pulsatile vehicle (middle) and constant CORT compared to constant vehicle infusion (right). Heatmap indicates merged replicates (N = 3), colour intensity indicates degree of fold change compared to VEH as shown within the -6 to 6 scale bar and genes clustered by log2 fold change values, hierarchically clustered by euclidean distances as indicated by dendrogram (far left). Number of genes differentially regulated are indicated under the appropriate lane. (B) VENN diagram indicates the number of genes differentially expressed by a single or combination of infused pulsatile / constant CORT or vehicle comparisons, as observed within the heatmap (A). (TIF) [file pgen.1009737.s006.tif]

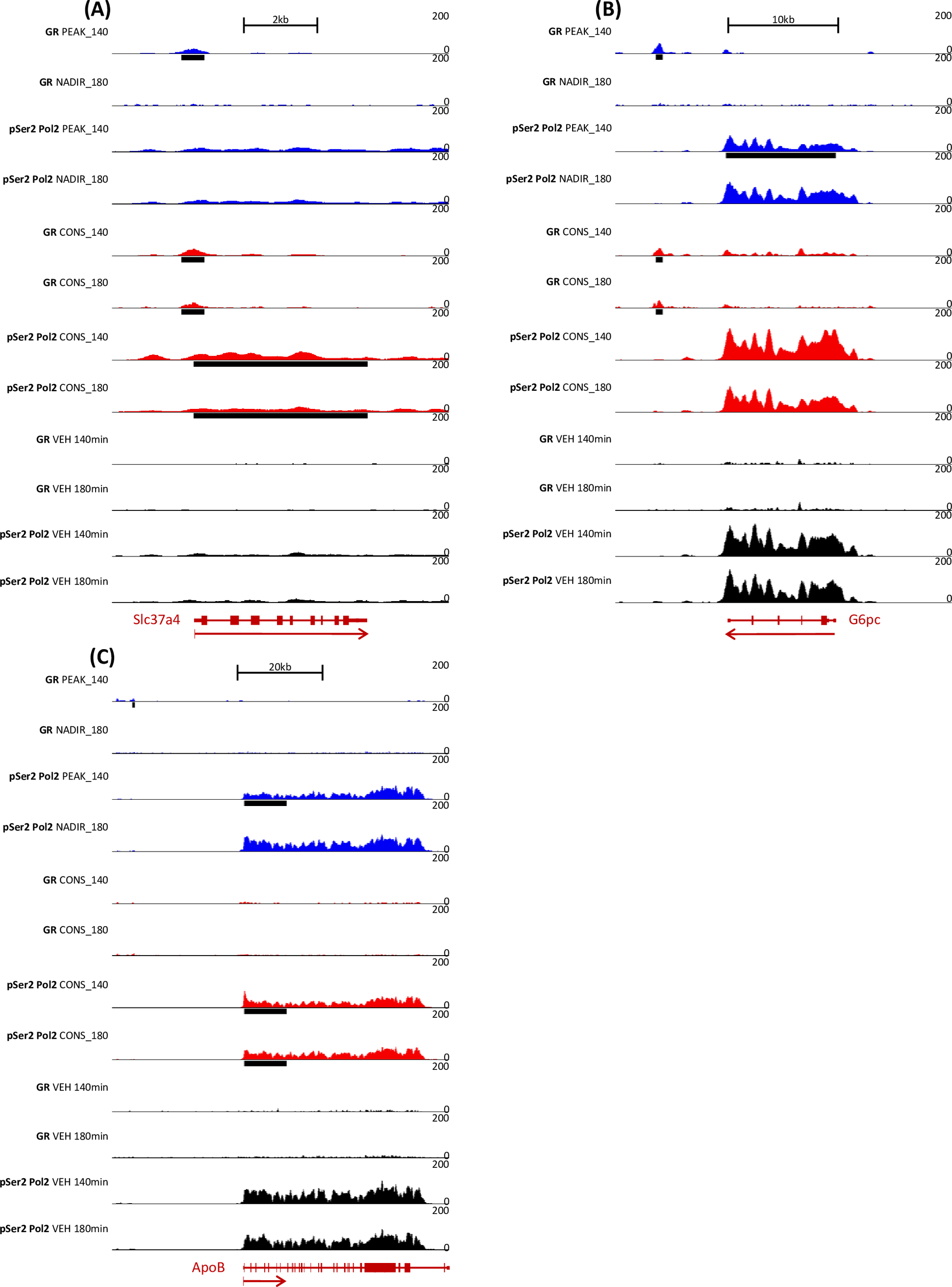

Supplement: S7 Fig — Both GR and pSer2 Pol2 tag density distribution was visualised using the UCSC genome browser within and extending from the TSS and 3’ end of three GC-regulated targets in response to PULS (blue), CONS (red) and VEH (black) after 140 min and 180 min. (A) GR enrichment was identified at the TSS of Slc37a4 in response to PLS_140 and CONS time points whilst pSer2 Pol2 intragenic enrichment increased in response to CONS only. Data visualised over a chr8: 48,716,099–48,725,227 region and y-axis set to 200. (B) Similarly, responsive GR enrichment was also detected 6 kb upstream of the G6pc TSS but pSer2 Pol2 intragenic enrichment was lost in response to PEAK_140 only. Data visualised over a chr10: 89,275,804–89,306,418 region and y-axis set to 200. (C) GR enrichment was identified in response to PEAK_140 only, 26 kb upstream of the ApoB TSS, whilst intragenic pSer2 Pol2 enrichment was reduced at PEAK_140, CONS_140 and CONS_180 for ApoB. Data visualised over a chr6: 33,145,813–33,224,922 region and y-axis set to 200. Data was merged from two replicates, normalised to 10 million tags and visualised using the UCSC genome browser within and extending from the TSS and 3’ end. Regions of CORT induced changes in enrichment to VEH are indicated by black bar below individual tracks. Scale bar is indicated at the top and locations of introns and exons (maroon blocks) according to Ensembl rn6 co-ordinates are indicated as transcribed from either the sense (left to right) or anti-sense strand (right to left) according to arrow direction (bottom). (TIF) [file pgen.1009737.s007.tif]

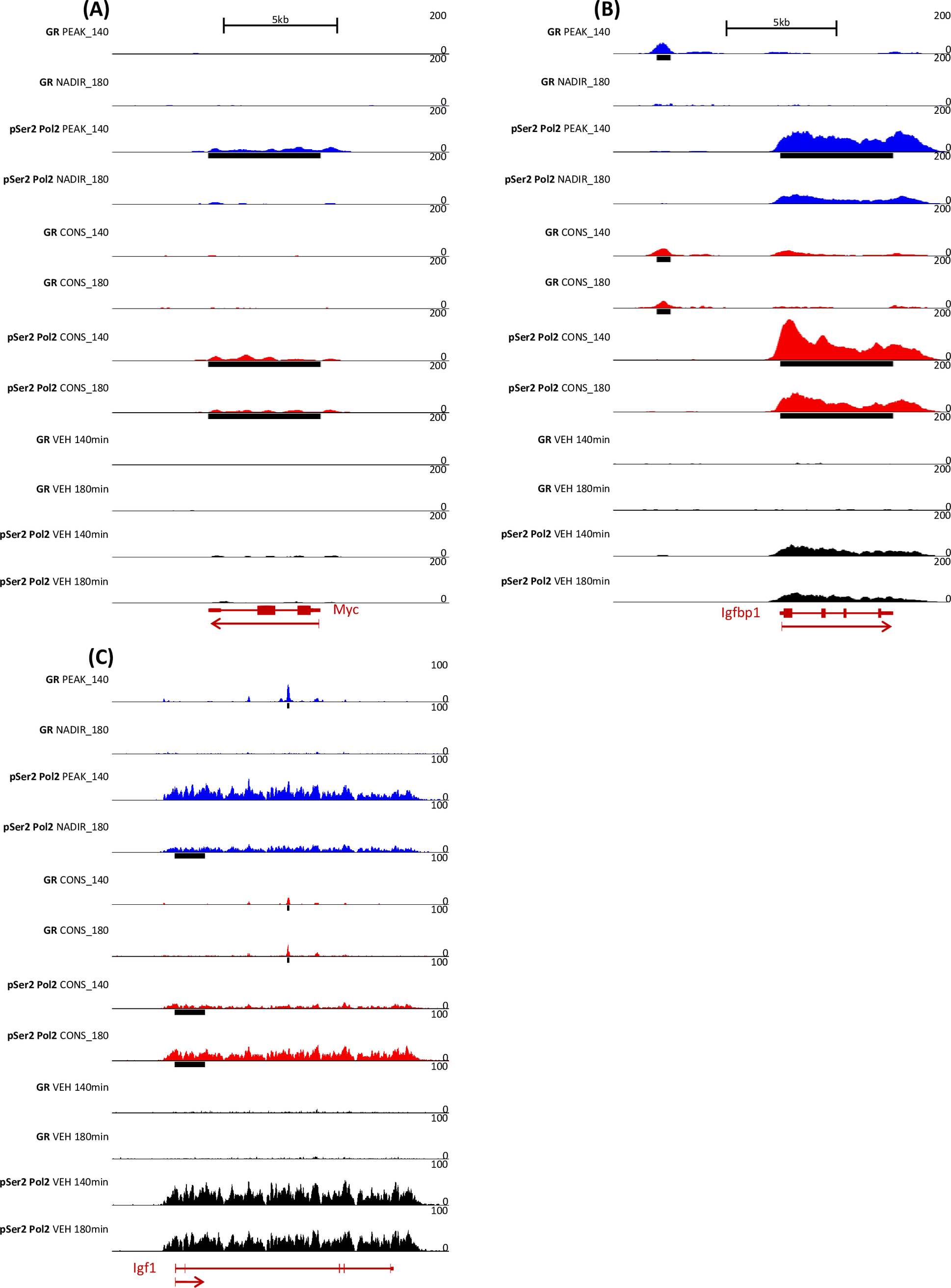

Supplement: S8 Fig — Both GR and pSer2 Pol2 tag density distribution was visualised using the UCSC genome browser within and extending from the TSS and 3’ end of three GC-regulated targets in response to PULS (blue), CONS (red) and VEH (black) after 140 min and 180 min. (A) No enriched GR sites were detected 50 kb up- or downstream of the Myc TSS, but intragenic pSer2 Pol2 enrichment was increased at PEAK_140, CONS_140 and CONS_180. Data visualised over a chr7: 102,581,386–102,596,166 region and y-axis set to 200. (B) CORT responsive GR enrichment was detected 5 kb upstream of the Igfbp1 TSS as was pSer2 Pol2 intragenic enrichment. Data visualised over a chr14: 87,441,155–87,456,358 and y-axis set to 200. (C) GR enrichment was detected 37.5 kb downstream within an intronic region of Igf1 in response to PEAK_140, CONS_140 and CONS_180 as was increased pSer2 Pol2 intragenic enrichment. Data visualised over a chr7: 28,393,595–28,505,212 region and y-axis for GR set to 200 and pSer2 Pol2 to 100. Data was merged from two replicates, normalised to 10 million tags and regions of CORT induced changes in enrichment to VEH are indicated by black bar below individual tracks. Scale bar is indicated at the top, y-axis set to 0–200 and locations of introns and exons (maroon blocks) according to Ensembl rn6 co-ordinates are indicated as transcribed from sense (left to right) or the anti-sense (right to left) strand as indicated by the arrow (bottom). (TIF) [file pgen.1009737.s008.tif]

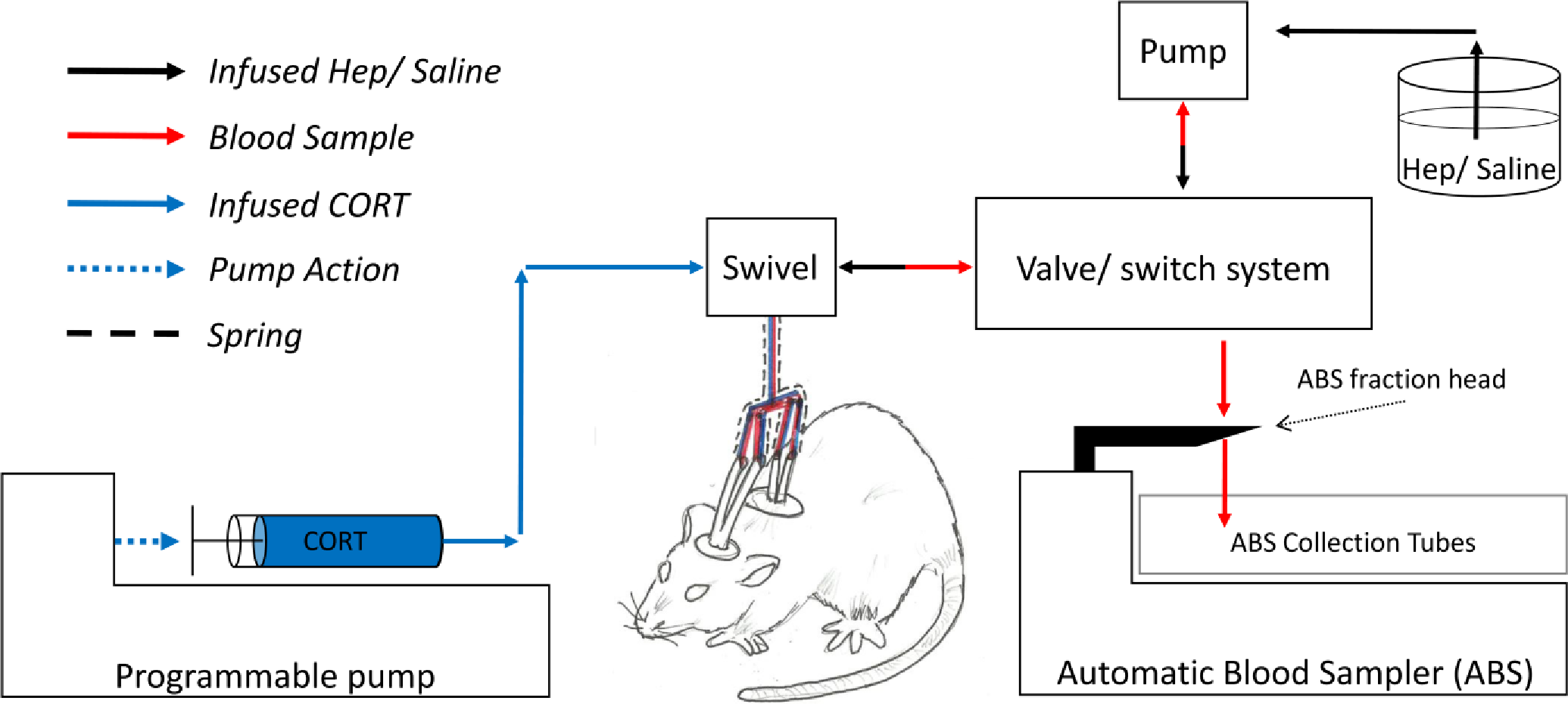

Supplement: S9 Fig — Rats implanted with intravenous cannulae were exteriorised either via craniotomy or vascular access “button” over the shoulders and attached to swivel to allow free movement. Solubilised CORT (blue) was infused via programmable pump (left). Automatic blood sampling system (right) withdrew blood (red) from the intravenous cannula through the swivel and valve/ switch system powered by a reversible pump. Once the required volume has been withdrawn, the pump is reversed, and the valves direct the flow to infuse the same volume of heparinised saline solution (black–top right) back through the intravenous cannula. Arrows denote direction of travel. (TIF) [file pgen.1009737.s009.tif]
