# Supplementary material for: Corticosterone pattern-dependent glucocorticoid receptor binding and transcriptional regulation within the liver
Source: PLoS Genet. 2021 Aug 10;17(8):e1009737. doi: 10.1371/journal.pgen.1009737 (PMC8378686; doi:10.1371/journal.pgen.1009737)
Supplement: Attachment — Submitted filename: 20210321_EditedReviewersComments_FlynnB.docx [file pgen.1009737.s018.docx]

Reviewer 1

The manuscript by Flynn and collogues builds on a number of studies from this lab seeking to characterize and understand the function of ultradian corticosterone/cortisol secretion in rodents and humans. The lab has published numerous studies showing that ultradian cort secretion manifests in dynamic GR occupancy, chromatin remodeling and gene expression. Most of these studies have been performed in cell cultures and little evidence is available from in vivo based model systems. In this manuscript Flynn et al uses a rodent model system to induce ultradian cort levels in serum and subsequently study genome-wide occupancy of GR in liver tissue. This is combined with genome-wide occupancy of elongating RNA polymerase II to probe for gene regulatory effects. Importantly, in agreement with previously published in vitro based data this study shows dynamic GR occupancy in response to pulsatile cort treatment. Interestingly, some regions of the genome show less pronounced GR occupancy in constant cort treatment compared to pulsatile cort treatment. Also, RNA polymerase II occupancy suggests that the impact on liver gene transcription may be differentially regulated in pulsatile versus constant cort treatment.

This study is of great importance in the efforts to understand the function of ultradian corticosterone/cortisol secretion in vivo. However, although the experimental setup is sound, the data analysis could be improved (see below). Also, the evaluation of gene expression using RNA polymerase occupancy could be improved by supported RNAseq analysis. Moreover, the study lacks mechanistic data or even suggestions to mechanisms implicated in constant versus pulsatile impact on GR occupancy and gene regulation. Suggested mechanisms may be more evident with additional data and data analysis.

Major comments:

1) Experimental setup. The authors use a very elegant experimental setup to mimic ultradian serum cort levels in a rat. As stated in the experimental procedures cort was given to adrenalectomized rats for 20min followed by a 40 min pause. This cort pulse was repeated three times over 3 hours and livers were collected at cort pulse peaks and nadir. However, it is unclear when during the day this cort pulse or constant cort was administered. As the authors nicely show in figure 1A-C ultradian cort secretion is most pronounced at the light/dark transition during a circadian rhythm. Thus, to mimic ultradian cort secretion and study the impact on gene expression it is also important to consider the time of the experiment. Especially since GR response to cort and subsequent regulation of gene expression is highly dependent on when cort is administered (PMID: 30179226). The authors should indicate when during a circadian rhythm the cort pulses were induced and why this particular timepoint was chosen.

We thank the reviewer for highlighting this as such a relevant and interesting study. Whilst we were concerned with out-of-phase molecular clock interactions, we felt that as the rats were adrenalectomised for 5 days, with a 16-hour withdrawal of corticosterone supplemented drinking water, we will have minimised any glucocorticoid dependent regulation of circadian synchrony.

Whilst the adrenal glands are the primary source of corticosterone production and secretion, extra-adrenal sources have also been documented from other endocrine and non-endocrine tissues such as the thymus (Vacchio, M. S., et al. 1994, PMID:8195711), salivary glands (Ieko, T., et al. 2019, PMID:31379745), testicular Leydig cells (Maeda, N., et al. 2015, PMID:31379745) (Wang, G., et al., 2002, PMID:31379745), small intestine (Cima, I., et al., 2004, PMID:15596520) and skin (Nikolakis, G., et al., 2014, PMID:25056134) (Slominski, A., et al., 2013, PMID:23435015). Although it is unlikely these sources provide a significant effect on circulating glucocorticoid levels, we were keen to remove all sources of endogenous corticosterone as this was essential to our experimental model. We therefore elected for a time point that coincided with the physiological circadian nadir of plasma glucocorticoids (nocturnal animals such as the rat is between Zt 13-16)

Additionally, the highlighted study used dexamethasone to induce glucocorticoid receptor activation. As dexamethasone has a much-prolonged half-life within blood serum of approx. 3hrs (Nicolaides, N. C., et al., 2018, PMID:25905379) than corticosterone at approx. 8-9min (Windle, R. J., et al., 1998, PMID:9449609), the use of either steroid could have very different transcriptional effects, as evidenced by Stavreva, D., et al (2015) (PMID25677181) where dexamethasone was a stronger repressor of gene transcription compared to corticosterone which induced a larger number of genes. Thus, drawing comparisons can be difficult and inherently flawed.

Line 430-431 within the method (subsection ‘Infusion Profiles’) has been edited to accurately state the time of experimentation and brief rationale.

2) ChIP-seq against GR in constant versus pulsatile cort conditions demonstrates a clear difference in GR occupancy under these two different conditions. How does this compare to a pool of GR ChIP-seq experiment using livers isolated around ZT12 during a circadian rhythm? In other words, how does the identified GR binding sites in the pulsatile/constant cort treatment compare with a normal physiological situation?

To our knowledge, GR binding within the liver of adrenally intact rats has not been assessed via ChIP-Seq methods. Recently, Kalvisa, A., et al., (2018) (PMID:30532187) published data within adrenally intact, 10-week-old C57BL/6N mice in response to either feeding or starvation at Zt12, followed by euthanasia at Zt14. Whilst this data is from mice and taken at a timepoint after the peak circadian corticosterone secretion, a degree of comparison can be made. Interestingly, genome browser shots from the Kalvisa paper are comparable to our data, with similar enrichment peaks (in both shape and height) for Pck1, Angptl4 and Tat. Whilst we appreciate that this is not the perfect comparison, we believe it provides evidence that our corticosterone replacement method reproduces similar GR ChIP-Seq enrichment profiles to those produced by endogenous adrenal corticosterone, albeit from a study performed in mice.

Additionally, whilst ultradian rhythms are highly conserved within mammalian systems, the peak and nadir of each pulse are not synchronised between individuals. Therefore, it would be flawed to directly assess data derived from maximal endogenous corticosterone levels in comparison to our infused patterns, particularly at a single time point. Furthermore, whilst we can ascertain circadian and ultradian corticosterone secretory profiles with high resolution blood samples collected every 10minutes using our automated blood sampling system, introducing timed euthanasia and tissue collection would most certainly induce a stress response in the rats which would be a confounding factor in these experiments. Nevertheless, we understand the reviewer’s point. We had also considered this in the design of our study, which is why we showed how our maximal replacement corticosterone peaks (Figure 1C,D) compared to endogenous maximal levels (Figure 1A,B).

3) The clustering shown in figure 2G suggests that GR occupancy is reduced in constant 140min cort compared to 140min pulsatile cort at about a half of the GR binding sites. Have the authors performed separate de novo motif analysis of these seemingly pulsatile specific GR binding sites and GR binding sites not occupied by GR under constant cort treatment? Also have the authors compared the GRE and half-GRE (referred to as AR half-site) motif score of these two types of GR binding sites?

Identification of *de novo* motifs within Figure 2G was assessed from all GR binding sites that were significantly enriched to VEH in response to PEAK_140, CONS_140 and CONS_180 time points. The analysis was done in this manner to identify the most prominent and statistically significant motifs and therefore identify the most likely co-factor interactions with GR. This method reduces the risk of misinterpretation and potentially biasing over-representation of consensus sequences in groups with a low number of binding sites. Co-occurrence of the top 6 most significant motifs was assessed in response to different patterned infused CORT time points (Figure S4B) and little change was noted, indicating no CORT pattern directed motif interactions.

To address the reviewer’s question, we have performed additional analysis. Binding sites were split by PEAK_140 enriched and PEAK_140/CONS_140 enriched as requested, and further *de novo* analysis applied to identify the occurrence of over-represented sequences according to the same methods mentioned in the manuscript (Table R1). Out of the 6 most significant short consensus sequences for both groups of GR binding sites, a motif complimentary of a GRE was top (Table R1A,C). Other motifs identified in both lists of sites was HNF1b which was ranked 6^th^ for PEAK_140 sites (Table R1A) and 10^th^ most significant for PEAK_140/CONS_140 (Table R1B). Additionally, NR2F2 which was ranked 2^nd^ for PEAK_140/CONS_140 sites (Table R1C) and also ranked 8^th^ for PEAK_140 sites (Table R1D). It should be noted however, that these sequences were matched from different databases.

Despite the motifs selected as most complimentary (highest ratio score) within either group, there were instances where motifs could be matched to a less complimentary motif selection. From PEAK_140 sites, CEBPE (ratio score 0.96) (Table R1A) had a sequence similarity with the PEAK_140/CONS_140 identified CEBPD motif (Table R1C,B). Looking through the list of potential consensus motifs for this sequence, CEPBE was ranked the 3^rd^ most complimentary, reducing the ratio score from 0.93 to 0.86 (Table 1B, red italicised text). Similarly, the CEBPD identified PEAK_140/CONS_140 motif (Table R1C) was ranked the 5^th^ most complimentary sequence for the PEAK_140 identified CEPBE motif, reducing the ratio score from 0.96 to 0.89 (Table R1D, red italicised text).

Similar observations were made for the PEAK_140/CONS_140 identified NF1-halfsite motif (Table R1C) with NFIC (Table R1D, red italicised text). The PEAK_140 identified STAT5 (Table R1A) has the potential for misinterpretation with PEAK_140/CONS_140 STAT1 motif (Table R1B, red italicised text), however this was for a differing database motif sequence.

Interestingly, this analysis did identify 2 motifs from each group of sites that may be over-represented independently. Within PEAK_140 sites PPARα (from liver ChIP) and FOXM1 (Table R1A) were identified as well as an AR-halfsite and Foxo3 motif for PEAK_140/CONS_140 sites (Table R1C). Whether these are representative of co-factor interactions with GR would require further validation and it becomes increasingly difficult to confirm whether they are similarly CORT pattern dependent as observed with the GR ChIP-Seq data. As this study was not designed to address these questions, despite its promising detail and highly intriguing results, we did not feel it appropriate to add to our submission without further investigation. We however thank the reviewer for their suggestion.

Regarding the GRE and AR half-site motif, we noticed a (%) typographical error with the ‘Score’ column and may have mislead reviewers (Figure 2K). This has been corrected. The GRE ratio score was highly confident at 0.92 whilst the AR half-site (which indeed is highly similar to the GRE half-site motif) was lower at 0.76. As this similarity score was reduced and the GRE half-site motif was not listed in the top 10 most complimentary matched motifs, we were not confident describing this *de novo* sequence as a GRE half-site, despite our similar suspicions. Finally, we found no particular distribution of GRE motifs (full or half) dependent on a corticosterone infused time point or association with another motif.

4) GR occupancy of chromatin does not necessarily lead differential regulation of enhancer activity. To determine if pulsatile and constant cort leads to difference in enhancer activity the authors should measure H3K27Ac at the identified GR binding sites. Alternatively, they could quantify RNA polymerase II at these sites as a proxy of enhancer RNA synthesis which correlates well with differential enhancer activity. Or total RNA-seq could provide insights to synthesis of eRNA.

Unfortunately, to collect data on the H3k27ac status at key GR binding sites, more liver tissue was required and there were serious delays accessing our research facilities due to the COVID-19 pandemic. We therefore apologise but are unable to produce this data.

We have assessed pSer2 Pol2 enrichment across the GR binding sites identified within this study as suggested, using methods similar to those used within the manuscript. The only change was total tags < 10 for each region were removed prior to DESeq2 analysis and results normalised to total counts within DESeq2 matrix.

We discovered a greatly reduced total of 237 sites (7.7 % of total GR sites) were differentially enriched for pSer2 Pol2 in response to both PULS and CONS across time points (Figure R1A). Interestingly, within the 237 sites we observe transient occupancy in response to PULS, with 106 sites (44.7 %) regulated at PEAK_140 compared to just 20 at NADIR_180 (8.4%) (Figure R1B). Comparable in dynamics to both GR as well as intragenic pSer2 Pol2 enrichment changes (Figure 2G & Figure 3A). In response to CONS, CONS_140 regulated 204 sites which was most of all differentially regulated sites (86.1 %) and two-fold the number to PEAK_140. Whereas CONS_180 was reduced at just 79 (33.3 %). As the changes in GR dynamics within this cohort of enriched sites is limited, this is difficult to assess without further investigation.

This data was not included within the manuscript as the purpose of the study was not to identify eRNA activity and the design limits our ability to fully explore this question or validate these results. It is however a very interesting question and we hope this goes in some way to satisfy the reviewers curiosity.

5) In figure 3 the authors show results from quantification of RNA polymerase II ser2 phosphorylation ChIP-seq. The quantification is performed at intragenic regions of the genome. Thus, referring to genes. They refer to these genes as sites or regions in figure 3 (panels A and F). Would be more appropriate with a consistent reference to genes instead. A consistent reference to genes may also be more appropriate in the text describing the figure. Here the intragenic regions are referred to as region, sites or genes. Would be most informative to consistently call these regions genes.

We appreciate the reviewer’s point and have accordingly modified the figure and text.

6) It is clear from figure 3A that the difference between pulse and constant cort is predominantly observed for repressed genes, while induced genes seems more alike. Yet, the comparison in figure 3F (now 3E) does not discriminate between induced and repressed genes. It would be informative to split figure 3F into induced and repressed genes.

Splitting 3E into up- and down-regulated genes would complicate the UpSetR graph and become very difficult to interpret. Figure 3D provides information of the number of genes and RNA that is either increased or decreased in response to CORT and we have added further bar charts (Figure 3C) which are also split in this manner. We hope these elements within the figure are an acceptable alternative to this request.

7) Unclear what is plotted in figures 3B-D (Figure S3B) and what information this specifically provides.

Any genes which had a FC >1.5 to VEH were then compared for FC >1.5 between 140 min and 180 min time points in response to either pulsatile or constant corticosterone infusions; labelled as “time dependent”. Similarly, the same differentially regulated genes to VEH were compared for FC >1.5 between pulsatile and constant corticosterone infusion at 140min or 180min; labelled as “pattern dependent”. This was to identify targets that were differentially regulated not only to VEH infusion but were significantly different between the differing patterns of corticosterone infusion. Finally, both constant time points were compared to NADIR_180 to highlight how the loss of ultradian pulse nadirs, effects gene transcription.

We understand the reviewer’s comment and have moved B-D from the main figure to the supplementary (Figure S3B) so as to not confuse the narrative.

8) Induced genes by cort pulse (140 peak) and constant cort (140 cons) seems similar. What is the log2FC pearson correlation between these two conditions for induced and repressed genes? Could be illustrated by a scatterplot.

Data was split into induced or repressed genes in response to PEAK_140, as this group had a greater number of changes compared to CONS_140. Results were included regardless of adjusted p-value or log2FC value thresholds previously described in Figure 2.

The log2FC pearson correlation coefficients indicated a weak correlation between PEAK_140 and CONS_140 values for up- (0.316) and down-regulated (0.264) genes (Figure R4A,B). Similar values were also observed in response to spearman rank and Kendal methods as Shapiro-Wilk tests indicated non-binomial distribution of values (data not shown).

Interestingly, distribution of log2FC values were similar between PEAK_140 and CONS_140 for up-regulated targets (Figure R4C), whereas PEAK_140 quantiles were greater for down-regulated genes (Figure R4D) compared to CONS_140.

9) It is very interesting that pulsed cort treatment leads to reduced elongating pol2 at genes while constant cort treatment leads to little or no reduction of pol2 occupancy. Is this reflected by RNA expression levels? In other words, does RNAseq using total RNA (e.g. ribo-zero prepared cDNA libraries) as input followed by intron quantification lead to the same conclusion? If RNA expression is reduced in both conditions this may indicate a different mechanism behind gene repression by pulsed and constant cort treatment.

We thank the reviewer for this idea, unfortunately we do not have liver from the 140min time point to assess RNA-seq in this manner. We have however been able to add data from liver RNA-seq at the 3hour timepoint and used analysis of exonic transcript expression data within the manuscript, expanding the study to include the nett effect on the liver transcriptome after 3 hours of pulsatile versus constant corticosterone infusion. Interestingly, the transient effect of greater gene repression we observed at 140mins with pulsatile compared to constant corticosterone, did not result in greater downregulation by the end of the timecourse and in fact we observed similar ratios of upregulated / downregulated transcripts irrespective of infusion pattern (Figure 3B). Strikingly however, constant infusion induced greater changes in both up- and down-regulation of genes, which is consistent with the findings of Stavreva et al (2015 PMID25677181).

In an attempt to answer the reviewer’s specific question, RNA-Seq analysis was also conducted within intronic boundaries via defining co-ordinates between exonic regions within 5’ and 3’ gene boundaries. Out of a total 1,461 transcripts differentially regulated to VEH, as before, CONS caused the greatest number of changes in 1,203 (82.3 %) transcript expression levels, whilst PULS was half this at 597 (40.9 %) (Figure 2RA). This is further represented by four-fold the number of differentially regulated transcripts by CONS alone (826) and not any other infusion time point compared to PULS (215) (Figure 2RB).

From the cohort of genes with a differentially occupied pSer2 Pol2 intragenic region, RNA-Seq identified 331 of these genes with differentially expressed transcripts (46.3 % of the 715 pSer2 Pol2 genes) (Figure R2C). Overall, PULS induced expression in 100 (14%) and down-regulation of expression in 57 genes (8%) whilst CONS induced expression in 127 (17.7%) and down-regulation in expression of 173 (24.2%) genes. Whilst overall there was little difference in the total number of up-regulated targets, there was decreased transcription in three times more targets for CONS than PULS.

We then assessed whether changes in pSer2 Pol2 intragenic occupancy was predictive of changes in intronic RNA-Seq data. The 140min ChIP-Seq time point was chosen for comparison as NADIR_180 had limited results. 123 genes had a result for both pSer2 Pol2 and RNA, of those 67 were expression was increased and 53 repressed, representing 54.5 % and 43.1 % respectively and accounting for 97.6 % of PEAK_140 pSer2 Pol2 regulated genes with a comparable change in transcription. Similarly, genes that were both differentially occupied by pSer2 Pol2 and differentially expressed RNA by CONS_180 totalled 166. 87 genes expression were increased and 76 were repressed, representing 52.4 % and 45.8 % respectively and accounting for 98.2 % of CONS_180 regulated genes with a comparable fold change transcription.

These data indicate that changes in the intragenic occupancy of pSer2 Pol2 are reflected in RNA expression at targets that were regulated in both Seq data sets. However, there does appear to be an increased number of targets up-regulated in response to CNS compared to PLS which is at odds with the overall ChIP-Seq results.

For details of these results, please see IntronRNAData_all_results.csv for all DESeq2 analysis results and IntronRNAData_significantresults.csv for results filtered by log2 fold change > 0.585 and adjusted p-value as well as the modified gtf file used to identify intronic regions, Rnor_6.0.92_includingBespokeIntronCoordinates_gtf.txt, within Supplementary Information.

10) In figure 3G pol2ser5-P distribution is plotted for all differentially regulated genes. Again, it would be informative to plot this separately for upregulated and downregulated genes. Maybe the pronounced peak shown in the CONS_140 condition is a result of stalled pol2 in that condition. Comparing with RNAseq data (mentioned above) could indicate if genes may be repressed as a consequence of stalled pol2.

As stated within the manuscript, these observations were made irrespective of treatment or time point and we did not intend to link these pSer2 Pol2 tag density profiles with either activatory or inhibitory transcriptional control, rather highlight evidence for the bimodal, promoter-pausing model of transcription as an interesting facet within the data.

There are limitations when splitting this type of analysis further, which is also why it was not attempted or included within the manuscript. The original analysis focussed on significantly differentiated genes with a coding region greater than 10 kb on the sense strand in response to any time point or infusion pattern. These limited restrictions were designed to avoid misrepresentation of tag density (due to potential bias genes of smaller gene coding regions). For this extra analysis, genes were further isolated from significantly differentiated genes in response to a single time point by either PULS or CONS and then split into up- and downregulated genes as requested. This reduced the number of genes greatly (table below) and distinctly impacts the power of the analysis.

Analysis within this cohort was visualised in the same manner as Figure 3F. Histograms of tag densities indicated tag pile up downstream of the TSS was present as in Figure 3F for both up- and down-regulated targets across all infused time points (Figure R3). Indeed, the greatest of these was found at CONS_140 for upregulated genes (Figure R3C). In conclusion, we cannot find evidence within this data that promoter-proximal pausing of pSer2 Pol2 is responsible for the inhibitive action observed within the ChIP-Seq data.

| Infusion Tx | Up-regulated | Incl. within analysis | Down-regulated | Incl. within analysis |
| --- | --- | --- | --- | --- |
| PEAK_140 | 147 | 46 | 406 | 160 |
| NADIR_180 | 24 | 7 | 80 | 31 |
| CONS_140 | 196 | 50 | 58 | 23 |
| CONS_180 | 139 | 42 | 141 | 47 |

11) It would be informative to also split genes/TSS plotted in figure 3H (Figure 3G) into induced and repressed genes. For example, figure 3Hi have an overrepresentation of repressed genes compared to genes/TSS plotted in figure 3Hii (Figure 3Gii). This may explain the differences seen.

Graphs from Figure 3G are split into induced (solid line) and repressed (dotted line) genes for each condition and the distance of the closest GR binding site in response to either PEAK_140, CONS_140 and CONS_180 is plotted along the x-axis. These graphs were used to illustrate that genes induced tended to have a GR binding site closer to the TSS than those that were repressed. These data do not consider whether there was greater pSer2 Pol2 accumulation (as observed in a promotor-pausing model of transcription) and does not explain those differences.

12) Figure 4A lacks information on the number of genes identified in each pathway. A dotplot like approach would provide this information together with a p value. Unclear how the z-score is calculated, and how this informs on negative and positive regulation of the pathway.

Z-scores and p-values are calculated from a list of multiple genes and their individual fold change and p-values. Therefore, a single z-score and p-value is calculated for each pathway. We have added the total number of genes used to calculate these values within the figure.

The z-score provides a scale of confidence whether the overall number of factors will either activate or repress a pathway and weighting that prediction against the fraction of studies that have published either activatory or inhibitory data, for each factor. Therefore, the z-score is a predictive value based upon whether there is a bias of gene fold inductions that are known to either activate or inhibit a pathway, according to a binomial distribution. It is recommended that any z-score ≥ 2 is predicted to work towards activation, whilst ≤ 2 works to represses a pathway.

Further information and formulae description have been added to the methods (line 543-549).

Minor comments:

13) Occupation is used frequently to refer to GR and pol2 binding to chromatin. Occupancy would be a more appropriate word.

Found and replaced.

14) Genome browser data shown in figures 4, S4 and S5 lacks labeling of the y-axis.

Added.

Reviewer 2

Reviewer #2: Flynn et al use an adrenalectionised rat model with cortisol (CORT) replacement in either a pulsatile plasma or continuous replacement pattern to assess the plasma pattern-dependent effects of CORT on gene expression in liver. The results confirm many earlier studies, by the present authors and others, in various cell culture models showing CORT pattern-dependent effects on genomics, epigenetics and gene expression (refs# 14, 25-28). Further, similar (albeit less comprehensive) CORT pulse-dependent effects were reported by the authors in an adrenalectomized rat model back in 2009 using a high CORT dose (Ref #14). The present study adds new information, most notably mapping to pattern-dependent binding sites for GR and pSer2 Pol2 at putative gene targets with potential physiological significance. However, there is relatively little by way of new mechanistic insight, and further, no effort is made to analyze or discuss the current findings in the context of earlier published studies, including the author’s own prior work on CORT/GR pattern-dependent responses.

1) New mechanistic insights are generally lacking but could be obtained by using the current set of livers to elucidate differences in chromatin states between pulsatile vs. constant CORT exposure, including differences in chromatin accessibility, (c.f., Ref. #28) and/or relevant activating and repressive histone marks. The addition of such data is considered critical.

We agree itt would indeed be interesting to look at differences in chromatin states. Our study however was already a very complex combination of novel physiological and molecular studies to establish a differential effect of oscillating hormone levels *in-vivo*. Our data now leads to further questions that will need to be addressed in future studies. We did in fact try to answer the reviewers interesting query and attempted to ChIP GR targets with a H3k27ac antibody. Unfortunately, the new animal studies needed were severely hampered by restrictions in our animal facility related to the Covid-19 pandemic and the antibody which had looked promising turned out not to be suitable for what we needed. Sadly therefore, this data will need to await new funding!

2) The major (new) findings here relate to differential gene targets of GR and pSer2 Pol2 between pulse and constant CORT. Two types of important supporting gene expression data are lacking: (A) Mechanistic Intronic RNA-seq analysis (or GRO-seq, etc.) is needed to determine if, in fact, there are significant differences in transcriptional rates between pulse and constant CORT for the differential pathways shown in Fig. 4ABC; and (B) Biological validation of result – RNA-seq of livers given Pulsatile vs. Constant CORT for a few days, to ascertain whether the differences in expression predicted by Fig. 4ABC are, infact, realized as ‘transcriptional outcomes’.

(A) As detailed in our reply to Reviewer 1 (9), assessment of intronic RNA-Seq of livers revealed that despite the greater number of differentially regulated genes and an overly inhibitive effect at PEAK_140, CONS induced greater changes that were equally repressive and activatory for either infused pattern. Additionally, genes that recorded significant activation / repression of pSer2 Pol2 occupancy also recorded the same direction of fold change in expression of RNA. Indicating the pSer2 Pol2 dynamics are linked. We therefore conclude that the transient effects observed at PEAK_140 are not all realised as transcriptional outcomes and it is the pro-longed regulation of pSer2 Pol2 occupancy by CONS that exerts greater transcriptional effects, providing further evidence of the importance of patterned CORT exposure to transcriptional effect. This is such an interesting result we have included exonic RNA-Seq analysis to the manuscript in an attempt to determine the transcriptional outcome of each CORT infused pattern. This additional analysis greatly increases the impact of our findings and is particularly striking when one considers the greater GR binding and pSer2 Pol2 occupancy regulation observed at PEAK_140 to either CONS_140 and CONS_180. We conclude therefore that despite increased circulating levels of CORT and transcriptional regulation at 140 min, sustained circulating (albeit lower than at the PEAK_140 time point) dose matched CORT levels increases the degree of transcriptional change compared to a highly transient profile induced by pulsatile CORT infusion. Additionally, there is overlap in pathways between both the ChIP and RNA data sets with the exonic analysis, regulating lipids and triglycerides (Figure 4F). Additionally, CONS regulated a greater number of genes than PULS (Figure 4D-F) as well as regulating genes over-represented in fatty acid, lipid, glucose, and liver development pathways. As mentioned previously, the pro-longed regulation of pSer2 Pol2 occupancy is concluded to exert a greater transcriptional effect within key metabolic pathways, as observed in the RNA, indicating that as these pathways are associated with described metabolic effects of hypercortisolaemia, indicating CORT pattern rather than dose/ amplitude can differentially affect metabolic regulation with a potential impact on metabolic health.

(B) We thank the reviewer for their comment and agree this is an interesting experiment. The infusion model we present within this paper, however, was modelled according to maximal circulating blood plasma levels within the rat (Figure 1A,B) and was delivered over a 3-hour period so overall 24-hour circadian changes in corticosterone did not have to accounted for. Simply extending the infusion period to 24 hours for multiple days would therefore not be representative of endogenous patterns, particularly during the inactive period where corticosterone is at negligible levels and would require an entirely new, greatly dynamic infusion model would be required with both circadian and ultradian elements.

The aims of the study we present here were to identify whether GR recruitment to the genome was 1) dynamically regulated by the ultradian rhythm, 2) transcriptionally relevant and 3) altered in response to dysregulation of pulses by constant infusion of the same dose. Extending this infusion model over multiple days is inappropriate in its current form and does not address the papers aims. In particular, infusing the current patterns for several days without incorporating circadian variation would induce a Cushings syndrome type of hypercortisol state in the rats. While interesting, this is another question altogether, and therefore beyond the scope of this study. It is something we are currently attempting to address, comparing the effects of both a smooth circadian and pulsatile ultradian rhythm. However, the data in this study was crucial to our understanding and design of these and other future studies so we would very much like to thank the reviewer for their comment and agree this is an extremely important experiment, particularly for assessing physiological processes.

For details of these results, please see IntronRNAData_all_results.csv for all DESeq2 analysis results and IntronRNAData_significantresults.csv for results filtered by log2 fold change > 0.585 and adjusted p-value as well as the modified gtf file used to identify intronic regions, Rnor_6.0.92_includingBespokeIntronCoordinates_gtf.txt, within Supplementary Information.

3) All raw high throughput data sets should be uploaded to GEO or another such public database, and accession numbers with reviewer access are needed. In addition supplemental Excel files with annotated, processed data listing all ChIP-seq peaks, peak intensities, differential sites, etc. should be provided.

Data has been added to submission.

4) Manuscript needs to be carefully edited to revise or clarify/explain apparent discrenpancies.
For examples:
a) Constant CORT levels, on lines 131 vs. line 146.
b) Light cycle times: line 138 vs. line 446.
c) Doses of CORT are unclear, and are listed in various inconsistent/inappropriate units for dosing: micromolar (line 141), rate in ml (line 143).
d) percentages referred to in text (lines 242-243) not explicity seen in the values shown in the associated Table in Fig. 3E (Figure 3D); kb units in text (line 289) vs. log 2 bp shown in Fig. 3H.

(A) These have been edited for more details as requested (lines 138-139 and 842).

(B) This was an error as the rats we collected the continuous endogenous circulating blood plasma corticosterone levels from were under 12:12 lighting, whereas our corticosterone replacement study was done under 14:10 lighting. It should be noted, this does not affect maximal circulating endogenous corticosterone levels and would therefore not impact our model. The methods have been edited to reflect this. (Lines 405-406)

(C) these have been clarified (line 837)

(D) Use of percentages has been removed (lines 202-205); this has been corrected (line 255).

5) How does CORT infusion, even in the controls (line 455) impact the basal level of CORT and the background of GR binding sites.

Whilst the adrenal glands are the primary source of corticosterone production and secretion, extra-adrenal sources have been documented from other endocrine and non-endocrine tissues such as the thymus (Vacchio, M. S., et al. 1994, PMID:8195711), salivary glands (Ieko, T., et al. 2019, PMID:31379745), leydig cells within testes (Maeda, N., et al. 2015, PMID:31379745) (Wang, G., et al., 2002, PMID:31379745), small intestine (Cima, I., et al., 2004, PMID:15596520) and skin (Nikolakis, G., et al., 2014, PMID:25056134) (Slominski, A., et al., 2013, PMID:23435015). It seems doubtful however that these extra-adrenal sources of glucocorticoids play any significant role in contributing to plasma levels of glucocorticoids and it has been well documented within our group and others that adrenalectomy reduces circulating CORT to negligible levels, as well as data from intra-tissue by micro-dialysis sampling, which are the same as within both our VEH and NADIR_180 infusion groups.

Within our data, we identified 50 binding sites which had significant tag density increase in response to VEH compared to PEAK_140 (Figure 2G) that had tag distribution indicative of real binding events (Figure S2F-J). We can therefore not rule the possibility of glucocorticoid receptors at these sites, as we refer to in the manuscript (line 170-174).

GR affinity for glucocorticoid ligand is relatively low compared to other glucocorticoid mediated nuclear transcription factors, such as the mineralocorticoid receptor (Reul, J, et al.,1987, PMID 3443807). Consequently, GR is expected to have greatly reduced binding to DNA during both circadian and ultradian nadirs. We therefore were unsurprised to find negligible GR binding within our NADIR_180 condition and during the VEH infusion. To investigate if there are any indications of basal GR binding, normalised tag count distribution and frequency was assessed (Figure R5C,D) within significant GR binding sites identified within our analysis (Figure 2B). What was apparent, particularly from the frequency graphs (Figure R5D), was there are two populations of tag counts in response to both VEH and NADIR_180. The first consist of sites with 0 tags, which we would describe as confidently non-GR sites. The second population, however, returned tag counts that peaked ~2.5 for VEH and ~3.5 for NADIR_180 tags (log2). Whilst both these were distinctly reduced from CORT mediated tag increases, we cannot currently explain these basal tag pileups, except to simply state ‘Tag density distribution plots support these as regions of enrichment indicative of real binding events (Figure S2F-J), although a mechanism explaining how GR binding can be greater in adrenalectomised rather than in CORT treated rat liver is currently not well understood. ‘

6) ChIP-seq data appear to be based on n=6 biological replicates which were pooled prior to sequencing (line 529). Is that true? If so, it seriously comprises the ability to know how reliable the differential peaks are.

We apologise for the confusion and have now made it more clear in the methods, describing exactly how we pooled replicates and analysed the data (lines 486-491)

7) Lines 223-227. Authors should consider the possibility that combinatorial patterns of the 6 motifs in Fig. S3B are differentially associated with the 3 types of peaks. This can readily be done by clustering each set of sites based on the panel of motifs with appropriate statistical analysis.

We attempted to address this within the manuscript and Figure S4A,B; we found no association between a PEAK_140, CONS_140 and CONS_180 infused time point and a specific motifs within GR binding sites. To confirm, we clustered sites dependent on which motifs were present and then compared those to the GR binding profiles (Figure S4C). Due to the lack of clustering observed within the GR binding sites across infusion time points, we believe there is no evidence of differentially associated motifs.

8) Fig. 1B. Comment how representative this is of the 6 profiles. Show the other 5 profiles in Supplemental.

Both Figure S1A,B are remarkably representative of Figure 1B and previously published ultradian profiles Windle, . R. J., et al. (1990) (PMID9751481)**.** All individuals produced similar overall circadian profiles, rising from basal at Zt 6-8, maximal secretion at roughly Zt 12 and reducing to basal levels by around Zt 22. It should be noted that Figure S1A,B,D profiles recorded a CORT increase between Zt 23-2 which we believe could be a technical complication of the automated blood sampling over 24-26 hour period, as we do not observe these levels in all individuals and at the beginning of the sampling period. Within each profile, there is ultradian pulsing to varying degrees, which is approx. hourly.

Figure S1A,B are highly representative of Figure 1B and in our experience, is common for individuals that sample very successfully. Figure S1C had reduced maximal secretion compared to other profiles, which we do occasionally observe within individuals. Additionally, low and high glucocorticoid responders (both basally and in response to stimulation) have also been described within humans, so whilst not as representative, these profiles are not unexpected within a group.

We believe Figure S1D has the capacity to reach similar maximal secretion levels, however sample collection was not very successful throughout the circadian peak period. This is a technical issue and was most likely dependent on the position of the animal and how this effected the indwelling cannula. Hence, potentially as the rat became more active, sampling became less successful.

It is difficult to discern the beginning of the circadian CORT rise for Figure S1E as no samples were collected prior to Zt 8. Additionally, whilst blood samples collected were not noted as being reduced from expected volumes, we cannot rule out the possibility sampling efficiency was reduced throughout and may have impacted on maximal CORT level measurements.

In conclusion, whilst it has been well documented both circadian and ultradian rhythms are highly conserved within mammalian species, ultradian pulse synchrony between individuals is variable. Despite this, Figure 1B and Figure S1A,B circadian and ultradian pulse dynamics are highly representative of each other. Which are also observable in Figure S1C-D despite technical sampling issues.

9) Fig. 2A-2F – Recluster across the sets of sites to illuminate subsets of sites. Y-axis label is unreadable.

Sites were re-clustered using k-means (4) and figure adapted.

10) Results should be discussed (compare and contrast) in the context of other liver biological systems that intersect with GR, and where epigenetic and transcriptional outcomes depend on the pattern of hormone stimulation, such as Growth hormone-activated Stat5 signaling to metabolic genes.

Notably, there is some evidence for pattern dependent differential transcriptional regulation in other pulsatile hormone systems. For example, pulses of growth hormone differ significantly in pattern between males and females (Jaffe, C., et al., 2001, PMID: 12376329), with distinct differences in GH-regulation of the liver transcriptome described in rodent studies (Choi, H., et al., 2000, PMID: 10984246). Additionally, growth hormones and GCs have been shown to have a synergistic role within energy homeostasis in the liver via targeted activation of nuclear factors such as STAT5 and GR; and impairment of their interaction can dysregulate lipid metabolism and induce hepatic steatosis (Friedbichler, K., et al. 2012, PMID: 22031092 and Mueller, M. et al., 2012, PMID: 21725989). Their episodic and circadian elements are an area of great interest, however, we wanted to focus on the dynamics of CORT regulated action and did not therefore broaden our discussion into other hormonal signalling networks.

Reviewer 3

The manuscript entitled “Corticosterone pattern-dependent glucocorticoid receptor binding and transcriptional regulation within the liver” builds on work done previously in the Lightman and Hager labs concerning the effect of glucocorticoid secretion rhythms on binding of the glucocorticoid receptor (GR) to DNA and consequent gene regulation. The existence of a pulsatile secretion rhythm of corticosterone (cort) on top of an overall diurnal rhythm was described previously in rats, and an effect of this pulsatile rhythm on GR binding and gene expression in cell culture has also been described. This work represents a step forward as the study was performed in a whole animal (rat) with the observed differences in a relevant tissue (liver). The administration and monitoring of cort in a pulsatile fashion, and in measuring differences in GR binding and gene regulation at corresponding intervals is a laudable technical achievement, and represents an important advance in understanding the role of glucocorticoid pulses in biology. The finding that certain unfavorable metabolic pathways are preferentially regulated by continuous administration of cort may also help to explain how dysregulation of glucocorticoids or pharmacological administration of glucocorticoids results in metabolic issues.

The collection of data and processing, in general, are solid, as is the overall interpretation. The experiments are also well reasoned. The authors do not make a strong conclusion that pulsatile exposure to glucocorticoids will lead to a more favorable metabolic profile than continuous administration (for example by continual regulation of cholesterol or carbohydrate metabolism), but this is appropriate, as the data are correlative, with no validation of the findings. This reviewer is strongly impressed by the system, and the demonstration that it shows something consistent with known biology. The lack of a strong conclusion, a comparison with results in cell culture systems, and follow-up to validate the links between differential binding/gene regulation and biology reduces the appeal to a broader audience.

Although the data collection and analysis appears solid, I have a few questions.

1) The measurement of cort in circulation every 10’ minutes is great. However, does the concertation in liver fluctuate by precisely the same period, or is it slightly out of phase? Measuring the concentration of cort in liver tissue at harvest timepoints could resolve this issue. It could also answer the question of whether the liver absorbs cort exactly the same way whether it is administered in a pulsatile or constant fashion.

Glucocorticoids are highly lipophilic, crossing cell membranes readily and rapidly diffuse into all tissues within the body. Regarding ultradian phase synchrony within tissue, previously published experiments have measured ultradian corticosterone oscillations in intra-adrenal extracellular fluid (Jasper, M. and Engeland, W.,1991, PMID1951775), during acute stress induced increases and within our own studies on ultradian dynamics within hippocampal extra-cellular fluid (Droste, S., et al. 2008, PMID: 18356272 and Droste, S., et al. 2009, PMID: 19477935) as well as with simultaneous measurements in blood, subcutaneous tissue and brain tissues via micro-dialyses (Qian, X., et al. 2012, PMID22822164) in the rat. These studies compared the dynamics of freely circulating ultradian corticosterone within the blood to subcutaneous and hippocampal measurements and reported a high degree of synchrony. The liver is a highly vascularised tissue and the robust corticosterone associated increases in GR binding across all identified sites at PEAK_140 before complete loss of significant sites within NADIR_180 (Figure 2G), are indicative of oscillating corticosterone levels we detected within adrenally intact rat circulation (Figure 1C,D), fully supporting our concept of blood: tissue synchrony.

2) Venn diagrams showing the overlap of genes or binding loci rely on arbitrary cutoffs, and can sometimes be misleading, or tell an incomplete picture. A scatterplot cmparing the normalized occupancy of each region under two different conditions would answer questions like: 1) Is GR bound to all of the same sites in PEAK_180 compared to PEAK_140, just less so? The answer to this would be very different than PEAK_180 GR being bound to only a subset of PEAK_140 sites. 2) Is GR bound to all the same sites in CONS_140 and CONS_180, but with just a few changes in the uncertainty of occupancy? This would be more informative of the biology of GR binding than saying that CONS_180 binds more sites (which is a distinct possibility). In other words, scatterplots can show trends in the data that are obscured by simple Venn diagrams that may be informative. For example, it is my hypothesis (which could be wrong) that the PEAK_140 binding pattern is simply more intense CONS_140 because there is, at that time point, more cort in the liver. If a scatterplot showed this (and quantified by simple linear regression), this could then be tested by increasing the CONS dose to match the instantaneous concentration in PEAK to see if there is a difference in binding pattern.

Pooled by infused time point, total tag counts were normalised to 10 million and pile ups assessed within the 3,098 identified enriched sites (Figure. 2).

1) Linear regression analysis between PEAK_140 and NADIR_180 time points (Figure R5A) indicated a low correlation (0.52) with a very weak r^2^ value (0.27). These data indicate that changes in PEAK_140 and NADIR_180 time points are not correlative.

2) There was a strong correlation (0.79) and r^2^ value (0.62) between CONS_140 and CONS_180 time points, indicating correlative changes in GR enrichment (Figure R5B).

To investigate this relationship further, tag count (log2) dispersion and frequency was plotted. PEAK_140 median as well as 75^th^ and 25^th^ percentiles were moderately raised compared to both CONS_140 and CONS_180, whilst all three were distinctly raised to both NADIR_180 and VEH (Figure R5C). Interestingly, NADIR_180 percentiles were slightly raised compared to VEH. This is further illustrated by two peak populations of tag density frequencies, one of which counted 0 tags within binding sites, whilst the other population recorded sites with a peak frequency of tag counts ~2.5 for VEH and ~3.5 for NADIR_180.

We therefore describe this data in three parts. One, a proportion of enriched sites by PEAK_140, CONS_140 and CONS_180 are confidently un-bound in both NADIR_180 and VEH conditions. Two, there is a tiny degree of tag density changes in NADIR_180 and VEH tag counts. We therefore cannot rule out the possibility of GR at these sites, but due to the lack of confidence, we would describe these events as negligible GR enrichment. Thirdly, there is evidence of highly correlative tag counts across both CONS time points, which are slightly reduced to PEAK_140. Given the relatively low affinity of GR for the endogenous ligand corticosterone (Reul, J., et al., 1987, PMID: 3443807), and consistent with the known dose responsive effects of corticosterone for GR (Reddy, T., et al., 2012, PMID: 22801371, Ong, K., et al., 2010, PMID: 20351279, Simons, S., et al, 1992, PMID: 1324423, Mercier, L., et al., 1983, PMID: 6129134, Koubovec, D., et al., 2005, PMID: 16125839, Vedder, H., et al. 1993, PMID: 8467386) we would postulate this is a result of differing circulating CORT at these specific time points as the dose is matched per hour, fluctuating in a pulsatile manner over time for PULS but not CONS.

3) The authors do a beautiful job measuring GR occupancy and gene regulation (using Pol2 occupancy as proxy). The patterns of binding are actually fairly similar between PEAK and CONS, with PEAK having more peaks, but the expression patterns are wildly different – with pulsatile strongly biasing toward repression - yet the authors present no model as to how more or transient binding favors repression. Is the same behavior seen at early timepoints for GR in other systems? Does it suggest a kinetic model where the early effects of GR are largely repressive? This is a very interesting finding that is largely not discussed.

Transcriptional inhibitory and transactivatory dynamics in response to CORT stimulation have previously been reported within hippocampal samples from adrenalecomtised rats (PMID 16503919) as well as nerve growth factor-differentiated catecholaminergic PC12 cells (PMID17026526) by Morinsk, M. et al (2006). Affymetrix GeneChip data indicated that whilst mRNA transcripts were mostly repressed within 1hr of CORT stimulation, by 3 hrs they were mostly increased before returning to basal levels by 5 hrs. Indicating both short- and long-term mediated effects of GR activation by CORT. This is certainly very interesting when one considers the differences in transcriptional activity between PEAK_140 and CONS_140 and suggests these dynamics may be connected in mechanism and suggests GR mediated transcriptional effects are highly responsive to acute, repeated interaction with liganded-CORT-bound GR. Morinsk, M. et al. suggests a dynamic model where competitive-inhibitory GR binding to transcriptionally regulating sites could be quicker in effect than transactivatory GR mechanisms, such as co-activator recruitment and chromatin re-organisation. Whilst we cannot comment on a molecular mechanism, what we can suggest with the addition of the RNA-Seq data, is a model whereby acute exposure of CORT represses recruitment of the transcriptional machinery at the genome. However, as transcriptional activity returns to basal levels within 40 min (NADIR_180), it does not efficiently repress mRNA production to significant levels.

It must also be considered that this study uses a physiologically relevant dose which is many times lower than those used in most studies, including the two mentioned previously. Additionally, we investigated the 3^rd^ CORT peak within a mostly intact biological system *in vivo*, whereas Morinsk, M. et al. used a single exposure in a highly *in vitro* model. We would hypothesise that in those studies, GR mediated mechanisms may have been sensitised to stimulation and perhaps generate a larger transcriptional result but cannot comment further.

4) Related to the point above: Although there are caveats to such analyses, the authors make no attempt to correlate binding at specific loci with regulation of proximal or associated genes. This might help to explain the mechanism of why repression is favored by pulsatile administration whereas gene activation is favored by long-term administration.

We were hesitant to make these conclusions as whilst GR binding in close proximity of a TSS is often associated with increased transcription (as supported in our findings) and within a study reported by Reddy, T., et al. (2009, PMID 19801529), it does not always mean that GR binding site is regulatory without site directed mutational studies to confirm. This is further compounded when one discusses long-distance GR mediated effects as the confidence based upon the assumption that the closest GR binding site is regulatory, becomes increasingly reduced.

We would hypothesise that the general assumption and model of GR transactivation is correct within what are usually termed ‘promoter’ regions. GR binds either independently or as part of a co-factor complex, recruits RNA polymerase complexes via co-factor interactions or increased chromatin accessibility to the transcriptional start site, inducing gene transcription. Long-distance interactions of repression are much less well understood, however as far as we are aware a general mechanism of chromatin looping and re-organisation reducing accessibility of transcriptional complexes to the TSS is currently the most accepted model of regulation.

I have a few general comments to improve the manuscript.

1) I’m confused by the layout of the manuscript. The figure legends appear to be embedded within the results section. I don’t know if this is how PLOS Genetics requests it, but I was not always sure when I was reading a result and when I was reading a figure legend. I’d prefer the figure legend separate from the results text.

Figures and the text have been moved from the results section into its own as requested and according to PLOS Genetics requested structure.

2) The Figures, labels, and legends are not always clear:

a. Figure 1E – The ampersands, dollars and starts above the bars are not explained in the legend.

Added (lines 851-853).

b. Figure 2 – the scale for 2A is “0 to 5”. Zero to five what? Fold enrichment? Normalized enrichment? This is not clear. Also, the y axis label at the far right is unreadable.

Added and y-axis enlarged and description edited (lines 857-858).

c. Figure 2: Often, and in other Lightman/Hager papers, it has proven useful to order each binding region heatmap by normalized tag intensity, or to cluster the tag intensities in order to reveal patterns of difference between samples. By eye it appears that there are clearly different clusters (e.g. about 2/3 of the way up comparing CONS-140 to CONS_180).

If the reviewer is referring to Figure 2A-F, then these have been re-plotted according to k=4 means clustering. Figure 2G is clustered according to complete Euclidean distances.

d. I couldn’t find a reference to 2J in the text, and it is a confusing comparison. Is this needed? Perhaps it could be replaced by Figure S3B, which is a more informative figure?

This graph was used to support the heatmaps of differentially enriched GR binding sites between each CORT infused time point (Figure S3A), showing the degree of overlap between infusion patterns and which GR binding sites they enriched. Mainly to show that PEAK_140 induced a greater number of sites independently of CONS. Additionally, CONS induced the same sites across both time points in a pro-longed manner. This has been added to the text (line 161).

e. The sites are percentages at the bottom of Figure 3A-D are not explained in the legend, and are not intuitive to understand.

Description has been added to the legend (lines 886-887).

f. The lines in Figure 3G are unreadable – I can’t tell whether to accept the conclusion made in the text or not.

I am un-sure what lines the reviewer is referring to, however, we have endeavoured to increase the size and resolution of all these plots in the hopes this more readable.

g. Similar to Figure 2J, Figure 3F (Figure 3E) is confusing, and is only used in the text to say that the biggest group of genes is the PULS_140.

The UpSetR graph was used in this manner as we felt a Venn diagram would have been overcomplicated for our conclusion which is, as the reviewer states, that genes differentially enriched by PEAK_140 alone were greater than any other infusion time point. Whilst this is also shown by the heatmap, we felt the resolution does not convey the numbers as well as the bar graph does.

h. Figure 3H (Figure 3G) is pointed out as an interesting finding, but no conclusion is drawn, so it’s hard to know why it is interesting or what it might imply.

Our conclusion to this data was that there appeared to be a link between an overall close transactivatory association of GR binding sites as opposed to a long-distance inhibitory action on transcription. We address the reviewers concern in our reply to their previous comment and hope this adequately explains our approach to this data and its interpretation.

i. Figure S1 (Figure S3A,B) has no scale bar, though the legend says it does.

Added

j. Figure S2 is unreadably small, and when there are two samples in one plot it is not possible to tell which is which. If there are no limits on supplementary figure size, it would be advisable to make this much bigger with darker, easier to distinguish lines.

These have been increased

k. As noted above, Figure S3B (Figure S4B) is an important enough result to include in the main paper.

We thank the reviewer for this comment but feel with so much data already in the main paper that it fits better in supplementary. If the editor would like us to add to the manuscript we are prepared to do so.

l. Any graph (Figure 4D,E,F, S4, S5) should have an x-axis label and scale.

Scale is labelled at the top of each of these graphs and the x-axis boundaries is quoted in the text. Y-axis labels were missing and have been added if this is what the reviewer was referring to.

3) The terminology “pattern dependent” in reference to how GR binds or which genes GR regulates, is sued throughout the manuscript, including the abstract, but it is not clear to me what it means. I do not think that this phrase clearly communicates that some binding and some gene regulation is dependent on pulsatile versus constant cort administration, if that is the meaning.

We feel that the term pattern dependent is clear enough for readers to understand that it is the ultradian pattern of glucocorticoids we are referring to.

4) Line 80 of the intro – the authors claim that GC side effects can result from “even low doses for a prolonged period”. What is meant by low doses? Doses below the dose equivalent of what is secreted endogenously? How do low doses (e.g. 20mg) of prednisone compare to the endogenous levels of cortisol secreted? My back of the envelope calculation would indicate that this would represent a huge increase. The authors should be more explicit, as their manuscript relies on the idea that low dose administration would be at or below endogenous, pulsatile levels.

We apologise for this confusion. The reviewer is indeed correct and even doses of synthetic GC are in excess of endogenous levels and we did not mean to suggest otherwise. Our point was that we believe the associated side effects are not entirely dose mediated and are also due to the dynamics of their regulatory action. We have amended the text to better reflect this (line 87-88).

5) Line 116 of intro – though MetS is listed as an abbreviation at the end, it is not defined in the intro prior to its use.

As this term is only used twice, the abbreviation has been dropped. Thank you for pointing this out to us.

6) Line 124 – what is “circadian active phase”?

Terminology was changed to “maximal circadian CORT secretion” (line 131-132).

7) Line169 – The authors say that CONS_140 has a “slightly reduced number” of sites compared to PEAK_140. It has 1273 sites compared to 2658, which is less than half. That is not slightly reduced.

This has been changed to better reflect these numbers (line 156-157).

8) For NADIR_180, the authors find not significant peaks over vehicle control, though by eye the tag density is clearly greater. NADIR_180 and vehicle are different, as is evidenced when NADIR_180 is used as a control and fewer peaks are identified in PEAK_140. This may be a case of a fold change cutoff obscuring identification of a statistically significant increase in binding. Indeed, there is still a substantial amount of CORT in circulation at the 180 time point – one would think that GR should still be bound to some loci.

Fold change cut offs were set at > 1.5 and adjusted p-values < 0.05. Whilst these are arbitrary thresholds, confident assessment of enrichment outside of these limits is very difficult.

As shown within Figure 1E, CORT levels at the time of euthanasia in VEH rats were not significantly different to rats euthanised at NADIR_180. Despite the trend for increase in in NADIR_180 CORT levels, this is still in the range of what we would consider negligible given the sensitivities of the radioimmunological assay.

Interestingly, when we assessed GR tag count distribution and frequency in response to VEH and NADIR_180 at sites we identified as significantly enriched, we saw that whilst a distinct population were equal to 0, other sites did have an increased tag count whose frequency peaked at ~2.5 for VEH and ~3.5 (log2) counts (Figure R5D). These frequency profiles would indicate the potential for GR at these sites, particularly as there is a trend of increase by NADIR_180 compared to VEH. However, volcano plots of NADIR_180 fold change to VEH illustrate the extremely low confidence of these sites making it very difficult to describe these as anything but negligible enrichment of sequenced tags (Figure R5E).

Upon reflection, we agree with the reviewer and do not wish to rule out the possibility of GR interactions outside of these thresholds, therefore, we have adapted the language used within the text to better reflect these interpretations.

9) Line 199 – it is not clear whether the motif analysis in Figure 2L/M was performed on a single sample or across all samples. This should be clarified in the legend.

At the beginning of the section we define the regions used for motif analysis, “To identify determinants of liver-specific GR binding, as well as pattern-dependent co-factors, all significantly enriched sites from the GR ChIP-Seq datasets were analysed for over-represented *de novo* motif sequences.” (line 175-178)

Additionally, at the start of the appropriate section in the legend “Table of the top 6 significantly over-represented sequences within CORT-regulated GR sites to VEH control.” (line 871-872)

10) Lines 202-206 – the authors state that there is unliganded GR binding to chromatin. To make the claim that there is no cort, the authors would have to test for cort concentration in the liver even and show that there is none even in adrenalectomized rats. Cort is synthesized in other organs, such as the thymus (see the work of John Ashwell), in mice. It may also be in rats.

Please refer to our answer to the reviewers first point. Additionally, we accept the reviewers point and have removed the phrase from the manuscript (line 171-172).

11) Line 217/218 – CEBPA and C/EBP should be consistent.

The use of C/EBP in this case was because this was what the reference we refer to used as nomenclature and we wanted to be as accurate as possible when referencing another author. This has been altered in the text as requested (line 184).

12) Line 228-230. The Hager lab and others have never found evidence of the nGRE motif in GR ChIP-seq data sets. Only the Chambon lab has found this, with questionable in vitro support by the Ortlund lab. The notion that this nGRE is real has been debunked and should no longer be discussed as a motif that directs GR binding. Repeating it here gives it continued life.

This was included within the manuscript as it was a common question when discussing this data with peers. We however agree with the reviewer and have removed it from the manuscript.

13) The authors use Pol2 ChIP as a proxy for gene expression – this is great. Perhaps it would make the paper more readable, then, to refer to enrichment or depletion of Pol2 at *genes* rather than *sites* in order to be sure that reader stays cognizant that this is the conclusion you are trying to draw?

It is and we have replaced the terminology.

14) Line 315 – “patterned time points” – I don’t know what this means.

We apologise for the confusion and have attempted to make it clearer, “enriched at each time point for both infusion patterns to VEH” (line 910).

15) Line 360 paragraph – this paragraph is more interpretation than presentation of results, and should be moved to the discussion. Same with the paragraph after that. Also – line 372, it is just hard to follow the logic – are the authors saying that CONS administration of cort may impair VLDL transport due to persistent repression of ApoB? I think so, but it should be stated more definitively.

These have been moved to discussion as requested and yes that was what we were trying to describe and have attempted to make this clearer.

16) Line 384 – The author states that all of the binding present at PEAK_140 “dissociates completely” at NADIR_180. The data do not support this. A visual observation of NADIR_180 tag density versus vehicle (Figure 2) indicates that there is *some* binding at NADIR_180. In addition, there are >2,600 binding sites in PEAK_140 compared to vehicle, but only ~1500 in PEAK_140 when compared to NADIR_180. This discrepancy can most easily be explained by residual binding of GR at NADIR_180. This may be a case of fold change cutoff producing a misleading interpretation.

The text has been edited to reflect these observations as has the rest of the manuscript.

17) Line 402 – The author claims that CONS administration “activates” the cholesterol biosynthetic pathway. This is not supported by Figure 4. Figure 4 shows that pulsatile administration strongly represses the pathway, whereas CONS still represses, but more mildly. This is a “failure to fully repress”, not an activation of this pathway.

This is an error in the explanation that has been rectified within the text. “Notably, pulsatile CORT was significantly predicted to repress cholesterol biosynthesis at 180 min and a trend at 140 min, whilst constant CORT was only predicted to repress at 180 min with a weaker z-score by functional pathway analysis, a potential risk factor associated with hypertension, obesity, liver fibrosis and non-alcoholic steatohepatitis (NASH) (46–48)” (line 354-358).

18) Line 429 – “chronobiological” is used to describe the effect of fluctuating GR levels on GR binding and gene regulation. This term is most frequently used to describe circadian or diurnal patterns. As the authors are attempting overlay a functional consequence to an ultradian rhythm on top of this circadian rhythm, I find this term confusing.

Whilst I agree with the reviewer the term ‘chronobiological’ is often used to describe circadian dynamics, the word itself describes biological rhythms that that are cyclic and, as far as I am aware, without restriction on the period or frequency. I felt therefore this term was applicable to describing ultradian dynamics and their effects to the transcriptome, as are applied to overall circadian dynamics. To avoid this confusion, the sentence has been refined and am willing to remove the term if still deemed un-acceptable (line 387-388).

**Review Figures**

**Figure R1 pSer2 Pol2 occupation within significantly enriched regions for GR in response to CORT infusion.** (A) Visualisation of the fold change in enrichment of pSer2 Pol2 to VEH (left) within the 3,098 identified GR binding sites (right) significantly enriched in response to PULS and CONS at 140 and 180 min. (B) Visualisation of the 237 sites where differential fold-change to VEH in both pSer2 Pol2 and GR enrichment were reported in response to PULS and CONS at 140- and 180-min. Total number of sites is indicated bottom left of each heatmap, in response to each CORT infused condition (top of each lane) and the number of sites differentially enriched (bottom of each lane) are indicated for both pSer2 Pol2 (top left) and GR (top right) is indicated in both heatmaps. Fold change (log2) of enrichment compared to VEH is indicated by key (centre top). Fold change > 0.585 (log2) fold change and adjusted p-value < 0.05 were considered significant and results were clustered according to complete Euclidean distances as indicated by the dendrogram (left of each heatmap).

**Figure R2 Changes in intronic RNA expression levels in response to 180 min pulsatile and constant CORT infusions.** (A) Total number of changes in RNA expression levels in response to pulsatile VEH compared to constant VEH (VEH – left lane), pulsatile CORT to pulsatile VEH (Pulsatile – middle lane) and constant CORT to constant VEH (Constant – right lane). (B) Of those total changes, VENN diagram indicates transcripts that were differentially expressed in response to either a single condition, or combinations of the three. (C) Of the 715 pSer2 Pol2 differentially occupied genes (lanes 1-4, left to right), differential expression of their associated transcripts (lanes 5-7, left to right) are visualised by their log2 fold changes. Data was hierarchically clustered by Euclidean distances according to fold change (log2) values > 0.585 (adjusted p-value < 0.05) and colour intensity indicates degree of fold change as indicated by the scale bar between -8 to 8 (top left of each heatmap). Total number of genes differentially regulated is indicated bottom left and number of genes regulated in response to infused CORT time point under each lane as well as associated percentage in brackets.

**Figure R3 Tag density distribution from the TSS of differentially up- and down-regulated pSer2 Pol2 occupancy.** Tag density profiles for differentially up-regulated (blue) and down-regulated (yellow) pSer2 Pol2 occupied genes compared to VEH were plotted within a region -5 kb up- and 15 kb down-stream off the TSS (0 kb) in response to (A) PEAK_140, (B) NADIR_180, (C) CONS_140 and (D) CONS_180. Genes were limited to > 10 kb intragenic coding region on the sense strand.

**Figure R4 Distribution of pSer2 Pol2 occupancy fold-changes in response to 140 min pulsatile and constant CORT infusion.** Out of the 715 differentially pSer2 Pol2 occupied genes in response to CORT infusion, results were split into (A) up-regulated and (B) down-regulated fold changes (log2) to VEH and linear regression and Pearson correlation coefficients calculated between PEAK_140 and CONS_140 results and noted within plots. Distribution of these fold changes (log2) in response to PEAK_140 and CONS_140 fold change values was also visualised by box-plot for (C) up-regulated and (D) down-regulated results.

**Figure R5 Correlative analysis and distribution of differentially enriched GR tag densities in response to pulsatile and constant CORT infusion.** Linear regression and correlation coefficients were calculated for normalised tag counts at 140 min and 180 min within 3,098 differentially enriched GR binding sites (fold change > 1.5, adjusted p-value < 0.05) in response to (A) pulsatile and (B) constant CORT. (C) Boxplots and (D) frequency distribution of tag counts (log2) were plotted in response to PEAK_140 (red), NADIR_180 (blue), CONS_140 (green), CONS_180 (purple) and VEH (grey). (E) Volcano plot of fold-changes (log2) and adjusted p-values (log10) of GR enrichment in response to NADIR_180 compared to VEH. Fold changes > 0.585 and adjusted p-value < 0.05 are indicated by horizontal and vertical dashed lines respectively.

**Table R1 of the top 6 most significant *de novo* motif discovery results within PEAK_140 and PEAK_40/CONS_140 enriched GR binding sites.** Sites that were enriched to VEH by a fold change > 1.5 and adjusted p-value < 0.05 by PEAK_140 were separated into two groups in comparison to CONS_140. The first group was enriched pulsatile only (PEAK_140) and the second was by pulsatile and constant CORT infusion (PEAK_140/CONS_140) after 140 min. Both sets of sites were assessed for *de novo* over-represented consensus sequences and matched to known motifs. Tables present the top 6 most significant motifs identified from (A) PEAK_140 and (C) PEAK_140/CONS_140 sites. (B,D) Tables describes motifs identified within (B) PEAK_140/CONS_140 over-represented sequences and (D) in PEAK_140 sites that are similar to motifs within PEAK_140/CONS_140 and PEAK_140 sites respectively. The most complimentary motif score to the *de novo* sequence is indicated (black), but if a matched motif between the two lists was not highest ratio score, best match rank is indicated as well as ratio score to *de novo* sequence in brackets (red).
